# Endotracheal intubation in the ICU

**DOI:** 10.1186/s13054-015-0964-z

**Published:** 2015-06-17

**Authors:** Stephen E. Lapinsky

**Affiliations:** Mount Sinai Hospital, Toronto and Interdepartmental Division of Critical Care Medicine, University of Toronto, Toronto, ON M5G1X5 Canada

## Abstract

Endotracheal intubation in the ICU is a high-risk procedure, resulting in significant morbidity and mortality. Up to 40% of cases are associated with marked hypoxemia or hypotension. The ICU patient is physiologically very different from the usual patient who undergoes intubation in the operating room, and different intubation techniques should be considered. The common operating room practice of sedation and neuromuscular blockade to facilitate intubation may carry significant risk in the ICU patient with a marked oxygenation abnormality, particularly when performed by the non-expert. Preoxygenation is largely ineffective in these patients and oxygen desaturation occurs rapidly on induction of anesthesia, limiting the time available to secure the airway. The ICU environment is less favorable for complex airway management than the operating room, given the frequent lack of availability of additional equipment or additional expert staff. ICU intubations are frequently carried out by trainees, with a lesser degree of airway experience. Even in the presence of a non-concerning airway assessment, these patients are optimally managed as a difficult airway, utilizing an awake approach. Endotracheal intubation may be achieved by awake direct laryngoscopy in the sick ICU patient whose level of consciousness may be reduced by sepsis, hypercapnia or hypoxemia. As the patient’s spontaneous respiratory efforts are not depressed by the administration of drugs, additional time is available to obtain equipment and expertise in the event of failure to secure the airway. ICU intubation complications should be tracked as part of the ICU quality improvement process.

One of the highest risk procedures carried out in the ICU is tracheal intubation. Significant complications occur in up to 40% of cases, with severe hypotension occurring in 10 to 25%, severe hypoxemia in 25% and cardiac arrest in 2% [1–4]. The incidence of death or brain damage is far higher following complications of intubation in the ICU than complications in the operating room (OR) [5]. This higher degree of harm has been attributed to the patients’ pre-existing hypoxemia and to hemodynamic instability [4, 5], but lack of recognition of the differences between intubation in the ICU versus the OR may play a role.

Endotracheal intubation in the OR and ICU are different procedures, although this is not always appreciated. The OR intubation usually involves a physiologically stable patient in an optimal environment. In contrast, the ICU intubation usually occurs in an unstable patient often with a period of time (albeit sometimes brief) to allow for evaluation and planning, and in an environment not always ideally suited to airway management. A significant proportion of ICU intubations are performed by relatively junior trainees, with or without supervision [1, 3, 5]. Unlike the OR where the primary objective is the induction of anesthesia, in the ICU the procedural objective is to secure the airway as a life-saving intervention in a patient with respiratory failure.

Airway management guidelines developed for anesthesia have been applied to the ICU environment, although the ICU patient is very different physiologically, and induction of anesthesia with resultant apnea is potentially harmful in this patient group. The stable patient about to undergo elective surgery may safely experience 6 to 8 minutes of apnea before developing significant hypoxemia, if adequately preoxygenated [6]. The ICU patient is usually intubated because of a pre-existing oxygenation or ventilation abnormality. Preoxygenation is usually ineffective in raising their oxygen saturation [4], and they will desaturate rapidly on induction of apnea, in some cases in as little as 30 seconds. The patient with a marked metabolic acidosis requires an adequate minute ventilation to maintain an acceptable pH, which is lost during a prolonged apneic intubation.

The definition of a 'difficult airway' is vague, encompassing difficulty in bag-mask ventilation, multiple intubation attempts, inadequate glottic view and complications [7], and is influenced by new fiberoptic technologies allowing an improved view. Prediction of a potentially difficult airway is based on history and a bedside assessment of anatomy [7]. Given the different timeframe available for intubation of the ICU patient, the airway assessment developed for the OR may not be applicable to the ICU. Furthermore, these methods to identify a difficult airway are not highly sensitive or specific, and unexpected difficult airways still occur. These can usually be managed in the OR, with access to additional expert staff, specialized equipment, and with the 6 to 8 minute buffer time, none of which are generally available in the ICU. With patients in the OR, bag-mask ventilation can usually provide adequate ventilation and oxygenation. However, the ICU patient with significant acute respiratory distress syndrome has reduced lung compliance, making bag-mask ventilation less effective. It is not unexpected, therefore, that intubation with techniques inducing apnea will result in the high complication rates cited above.

Given these physiological and situational differences between ICU and OR intubations, a safe approach would be to consider all ICU intubations as 'difficult airways'. Difficult airway algorithms begin with a decision of 'forced to act', which necessitates an immediate best possible attempt, or 'not forced to act', where the main tactic is an awake intubation. The safest approach for an ICU intubation is to consider an awake attempt. This is in contrast to the current practice where the initial approach is almost always induction of deep sedation with or without paralysis (inducing apnea). A rationale given for the common use of sedation and neuromuscular blockade during intubation in the critically ill is that paralysis improves the rate of successful first or second attempts, and that the complication rate of intubation increases with multiple attempts [8]. This circular reasoning fails to acknowledge that the reason for complications with multiple attempts is, in fact, the presence of drug-induced apnea producing hypoxemia.

In some agitated patients the awake approach will not be possible. However, a remarkable number of ICU patients will accept intubation with little more than topical anesthetic - some sedation usually being provided by the accompanying sepsis, hypoxia or hypercapnia. Direct laryngoscopic view of the vocal cords has been shown to be possible in the majority of well patients with minimal sedation [9]. Awake intubation sometimes implies complex instrumentation with which junior trainees are not familiar (for example, fiberoptic bronchoscopy) and so awake intubation becomes the purview of the expert. However, there is little to be lost by an initial look in an awake and breathing patient with a conventional laryngoscope and a local anesthetic spray. Either the patient will be able to be intubated, or if they are a little resistant but the airway looks easy, a small amount of sedative may facilitate the procedure. If the airway appears impossible for that operator, the procedure can be abandoned awaiting expert assistance - the patient remains breathing and is no worse off than prior to the intubation attempt. Recent studies and commentary suggest a role for awake videolaryngoscopy in the patient with a difficult airway [10] - hopefully a practice that will soon migrate to the ICU.

## Conclusion

Let us work to eliminate this high complication rate of ICU intubation. Tracking of ICU intubation complications should become an essential quality improvement process. Intubation-related catastrophes in the ICU commonly occur after-hours and by staff with less experience [5], who continue to be taught the same methods that would be used in the operating room on physiologically stable patients and under expert supervision. Awake intubation is an accepted intervention in the patient with an anticipated difficult airway [10, 11] and should be the standard teaching as an initial approach when intubating the critically ill, particularly intubations by those with limited experience.

## Abbreviation

OR: Operating room
